# Food-induced changes of lipids in rat neuronal tissue visualized by ToF-SIMS imaging

**DOI:** 10.1038/srep32797

**Published:** 2016-09-06

**Authors:** Masoumeh Dowlatshahi Pour, Eva Jennische, Stefan Lange, Andrew G. Ewing, Per Malmberg

**Affiliations:** 1 Department of Chemistry and Chemical Engineering, Chalmers University of Technology, Gothenburg, Sweden; 2 National center for imaging mass spectrometry, Gothenburg, Sweden; 3 Institute of Biomedicine, Sahlgrenska Academy, Gothenburg, Sweden; 4 Department of Chemistry and Molecular Biology, University of Gothenburg, Gothenburg, Sweden

## Abstract

Time of flight secondary ion mass spectrometry (ToF-SIMS) was used to image the lipid localization in brain tissue sections from rats fed specially processed cereals (SPC). An IonTof 5 instrument equipped with a Bi cluster ion gun was used to analyze the tissue sections. Data from 15 brain samples from control and cereal-fed rats were recorded and exported to principal components analysis (PCA). The data clearly show changes of certain lipids in the brain following cereal feeding. PCA score plots show a good separation in lipid distribution between the control and the SPC-fed group. The loadings plot reveal that the groups separated mainly due to changes in cholesterol, vitamin E and c18:2, c16:0 fatty acid distribution as well as some short chain monocarboxylic fatty acid compositions. These insights relate to the working mechanism of SPC as a dietary supplement. SPC is thought to activate antisecretory factor (AF), an endogenous protein with regulatory function for inflammation and fluid secretion. These data provide insights into lipid content in brain following SPC feeding and suggest a relation to activating AF.

Antisecretory Factor (AF) is a 41 kDa regulatory protein acting on hyper fluid secretion and inflammation which is naturally secreted in all cells, plasma and other tissue fluids of mammals^1^,^2^,^3^,^4^,^5^. An endogenous increase of AF counteracts the clinical intestinal symptoms in patients suffering from inflammatory bowel disease^5^ and is also known to provide protection against raised intracranial pressure induced by experimental head trauma^6^. The “active site” of AF was found to be 8 amino acids located in the N-terminal part of this protein and this 8-amino acid peptide is responsible for regulating inflammation and secretion^7^. Interestingly, the level of plasma AF in human depends on body condition such that most plasma AF is inactive in physiologically balanced persons^8^,^9^,^10^. It has been proven that AF protein can be activated by either intestinal exposure to bacterial toxins or consumption of certain food components^10^. One of these dietary compounds is specially processed cereals (SPC), which has been shown to enhance active antisecretory factor (aAF) in plasma^10^,^11^. Correspondingly, a SPC-induced increase of aAF plasma levels was shown to reduce vertigo and recover hearing in patients suffering from Menieres disease^12^. In general, the clinical results obtained from research on using SPC-treatment for various diseases indicate that SPC-intake also might influence nervous tissues directly. Thus, intracranial pressure in the brain induced by an experimental head trauma was significantly decreased after four weeks of SPC intake in rats^6^.

Lipids are the main components of all mammalian cells providing the structural integrity necessary for protein function as well as serving as lipid anchors to bind proteins to the cell membrane^13^. Moreover, lipids function to store energy, and also serve as reservoirs of second messengers for cellular signaling^14^. In addition, the brain is the most complex organ in the body and mainly consists of lipids such that 50-60% of the brain’s dry weight is lipids^15^,^16^,^17^. Accordingly, as lipids are extensively involved in all cellular processes and tissue physiology, it is not surprising that abnormalities in lipid composition and distribution, and even the ratio of different lipids, are influenced by normal and abnormal biological functions^17^,^18^,^19^. Several studies have revealed the effect of perturbation and alteration of particular lipids on neurological disorders such as Parkinson’s, Alzheimer’s, Schizophrenia and multiple sclerosis^17^,^20^,^21^,^22^,^23^,^24^. Hence, lipidomics, which is the investigation, characterization, and structural analysis of lipids and their interacting moieties, is a powerful research tool in biological studies^15^,^19^. However, due to the extremely complex nature of lipids, there is a real need for global analytical, label free techniques with high chemical specificity, high accuracy and high sensitivity. A major innovation during recent years has been to develop new mass spectroscopic techniques spurring advances in lipid analysis^25^,^26^. One of them is the ultra-high surface sensitive technique of time-of-flight secondary ion mass spectrometry (ToF-SIMS) which has been rapidly recognized as a very useful approach for studying biological compounds particularly lipids in tissue sections and biological samples^27^,^28^,^29^,^30^,^31^,^32^.

Changes in lipid content might play a role in the mechanism of activation of AF. Laurenius and coworkers worked on this mechanism and suggested that activation of AF might be correlated to an SPC-induced unmasking of epitopes working as potential ligands for specific AF binding sites^33^. Consequently, we assumed that if this suggested mechanism is true, changes in lipid content of the cell membrane in the intestine is very likely in rats fed a SPC diet. In this regard, we recently studied lipid components in intestinal tissue followed by SPC diet^34^. Our findings showed obvious changes in the distribution of certain lipids in intestinal tissue from rats fed with SPC, positively supporting Laurenius work.

Some interesting studies have been done on the effect of a diet of SPC on aAF in the brain. A four-week dietary intake of SPC in rats has been demonstrated to counteract an increase of intracranial pressure in response to an experimental head trauma^6^. It has also been demonstrated that AF in the central nervous system (CNS) interacts with subcellular structures^35^,^36^. Again, if this is correct, then changes in lipid content of cell membrane in the brain are highly expected. For that reason, we were motivated to continue our investigation, this time on the lipid content of cell membranes in brain sections followed by a SPC diet.

In this paper, we used ToF-SIMS to investigate and compare the lipid content of cell membranes in brain sections of rats fed SPC with control rats fed a normal diet. Principle Component Analysis (PCA) was performed to discriminate between the two groups. A major point was to further understand the mechanism of AF activation of food-induced changes in the cerebellum and stem regions of the brain. We also aimed to evaluate if changes in separate regions of interest in the brain could be analyzed individually with PCA and if different regions show different types of lipids changing.

## Results and Discussion

### ToF-SIMS imaging of brain tissue after SPC feeding

**Figure 1:**
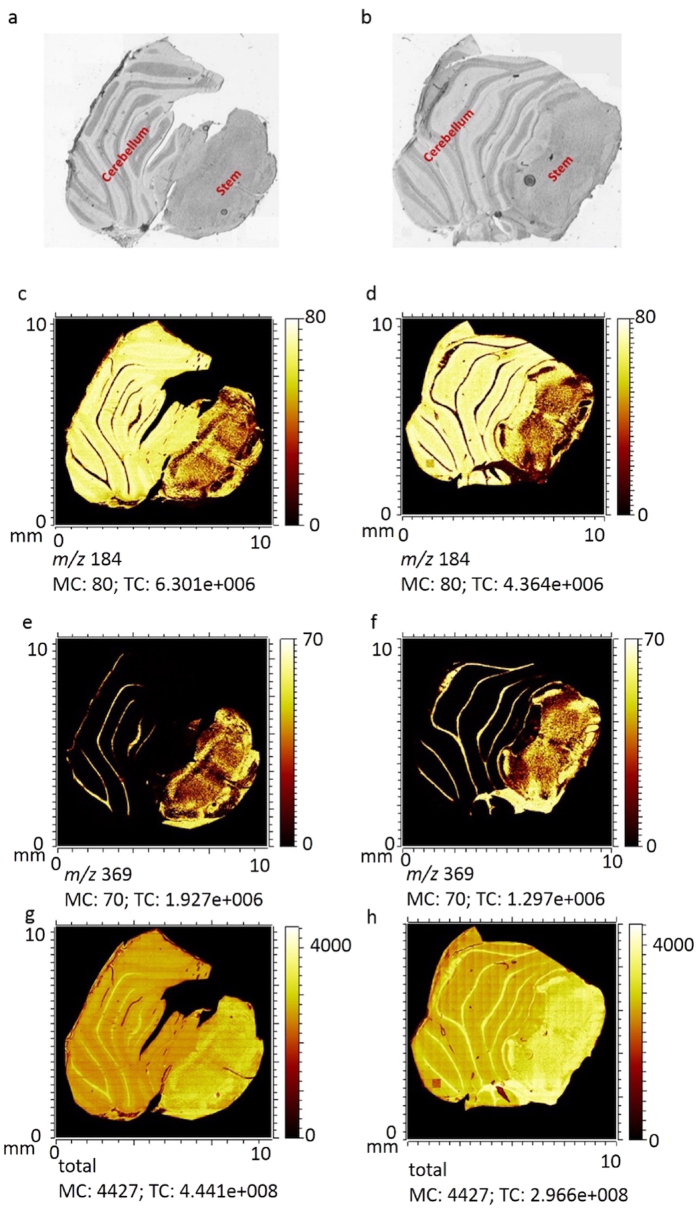
Optical microscopy images of freeze-dried rat brain sections brain section containing cerebellum and stem are shown in (**a**) control and (**b**) the SPC-fed group. ToF-SIMS images showing the spatial signal intensity distribution from positive ions are shown for m/z 184 (phosphocholine): (**c**) control and (**d**) the SPC-fed group; for m/z 369 (cholesterol): (**e**) control and (**f**) the SPC-fed group; and for total ions: (**g**) control and (**h**) the SPC-fed group; across an analysis area of 11 × 11 mm covering the complete tissue.

In the present study, we used ToF-SIMS imaging to investigate, image and localize the lipid content in rat tissue from the cerebellum and stem brain regions. Analysis was carried out on 3 different rats containing 15 sequential sections in both control and SPC-fed groups. Figure 1(a,b) show optical microscopy images of a representative freeze-dried section in control and SPC-fed groups used in this study, respectively, taken prior to measurement. This clearly shows the cerebellum and the stem regions of the brain. ToF-SIMS images of some single molecular species abundant in brain tissue are shown for positive mode in Fig. 1(c–h). The images of positive ions for *m/z* 184 (phosphatidylcholine headgroup [C_5_H_15_PO_4_N]^+^) as chemical species that localize to gray matter are illustrated in Fig. 1(c,d) for control and SPC-fed groups, respectively. Correspondingly, ToF-SIMS images of positive ions for *m/z* 369 (cholesterol ([C_27_H_45_]^+^) as the most abundant lipid species in white matter are shown in Fig. 1(e,f) as well as the total positive ion images in Fig. 1(g,h) for both groups of control and SPC-fed animals. Similarly, ToF-SIMS images of some negative ions obtained from the same control as well as SPC-fed tissue sections are shown in supplemental Fig. S1.

The optical images shown in Fig. 1(a,b) correlate well with the ToF-SIMS images of both positive and negative ions desorbed from the surface of the samples. This validates that ToF-SIMS is a powerful technique to localize, map, and image the distribution of surface lipid compounds within the complex anatomy of brain tissue sections. It also reveals that there is sufficient chemical information across the freeze-dried rat brain tissue sections to produce ion images that show the tissue structure and make it possible to distinguish between histological features such as gray and white matter, as well as the stem and cerebellum regions. However, no apparent changes in lipid distribution could be detected by comparing the ion images from the two different groups. Also, due to the huge data set containing a 453 (negative ions) and 696 (positive ions) peaks obtained from 15 brain samples per each group, it is time consuming and almost impossible to look at all ion images in order to recognize how different lipid species are changing from sample to sample. Therefore, to deal with the large data set, multivariate data analysis using PCA was performed on the spectra obtained from TOF-SIMS analysis to differentiate and discriminate the data from control versus SPC-fed rats.

### PCA data analysis to examine lipid changes in SPC-fed animals

**Figure 2:**
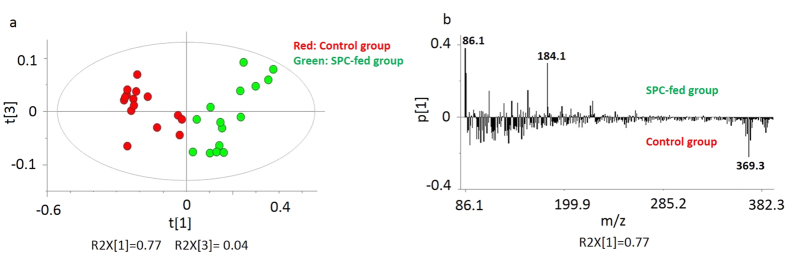
PCA analysis of positive mode data for the ToF-SIMS experiment for 3 rats imaging 15 brain tissues containing the cerebellum and stem regions for SPC-fed versus control samples. (**a**) Score plot of the first principle component (t[1]) vs. the third principle component (t[3]) from the spectra. (**b**) Corresponding loading plot of the first principle component (p[1]) representing the m/z peaks with strong impact on the separation between groups.

In order to compare and to investigate any changes in lipid content of the brain tissue sections, PCA was performed on all collected and normalized spectra obtained from ToF-SIMS analysis on 3 different animals from each group of control and SPC-fed with five technical repeats. In PCA analysis of the whole brain tissue in positive ion mode, no clear separation was observed using the first principal component (PC1: t[1]) versus the second one (PC2: t[2]) (data not shown), therefore here PC1 versus the third principle component (PC3: t[3]) was used and related score plot is presented in Fig. 2(a). Different color symbols were used to indicate control in red and SPC-fed in green colors. The groups are well separated and the model captures 81% of the variance in the data. Clearly, the changes in lipid content are sufficient to chemically differentiate the two groups. Examining the corresponding loadings plot of the first principle component (PC1: p[1]) in Fig. 2(b) reveals the masses driving this separation between two groups. The most specific biological masses among the highest loading peaks are *m/z* 86 (PC: [C_5_H_12_N]^+^), *m/z* 184 (phosphatidylcholine headgroup [C_5_H_15_PO_4_N]^+^), which are associated with the SPC-fed group and *m/z* 369 (cholesterol [C_27_H_45_]^+^), which is associated with the control group.

**Figure 3:**
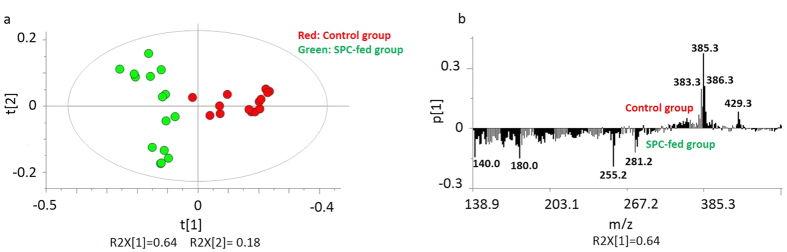
PCA analysis of negative mode data for the ToF-SIMS experiment for 3 rats imaging 15 brain tissues containing the cerebellum and stem regions for SPC-fed versus control samples (**a**) Score plot of the first principle component (t[1]) vs. the second principle component (t[2]) from the spectra. (**b**) Corresponding loading plot of the first principle component (p[1]) showing the m/z peaks with strong impact on the separation between groups.

Figure 3(a) displays the score plot of PC1 and PC2 from the whole tissue section in negative mode. Here, these two principle components again demonstrate relatively good discrimination between the control and SPC-fed rats covering more than 82% of the cumulative variance. The corresponding PCA loadings plot illustrated in Fig. 3(b) shows the peaks primarily responsible for the differentiation of the groups. The biologically relevant molecules are *m/z* 140 (phosphatidylethanolamine), *m/z* 180 (phosphinothricin), *m/z* 255 (palmitic acid [C_16_H_31_O_2_]^−^), *m/z* 281 (oleic acid [C_18_H_33_O_2_]^−^), *m/z* 385 (cholesterol [C_27_H_45_O]^−^) and *m/z* 429 (Vitamin E [C_29_H_50_O_2_]^−^). The control group is associated with *m/z* 385 (cholesterol [C_27_H_45_O]^−^) and *m/z* 429 (Vitamin E [C_29_H_50_O_2_]^−^), whereas the SPC-fed group is associated with *m/z* 140 (phosphatidylethanolamine), *m/z* 180 (phosphinothricin), *m/z* 255 (palmitic acid [C_16_H_31_O_2_]^−^) and *m/z* 281 (oleic acid [C_18_H_33_O_2_]^−^).

Despite of the separation between rats fed SPC and control rats, the data still suffer from variation within the groups resulting in scattered data points. This variation arises from several sources. One is related to the inherent biological variation existing in the different animals analyzed. This kind of variation is to be expected in our study since the data obtained here were from 15 brain samples from 3 different rats for each group. Even though the rats have a consistent genetic background there are still phenotypic variations remaining among individuals giving rise to the sample variation. A lot of attention was given to sectioning the tissue in a repeatable manner, yet there remains a variation in the sectioning of the brains within the groups. To minimize this we have chosen brain cross-sections through the cerebellum and the brain stem to achieve the highest possible similarity between consecutive sections. Another source of variation is instrument-related. A slight variation in ToF-SIMS spectra can be found even if homogenous solid surfaces are analyzed^37^. Here, ToF-SIMS was acquired over several days generating more instability and variation in the data which was unavoidable due to amount of samples. The storage time at −80 degrees in which changes might occur in the tissue sample can be another source of variation in the data. Yet, no samples were stored for longer than 3 months before analysis.

The brain sections were also analyzed based on the histological features i.e. by separating the data into regions of interest based on the stem and cerebellum parts of the brain sections to evaluate individual lipid changes in separate regions of interest. Figure S2(a,c) show score plots of PCA on positive ion data from stem and cerebellum tissue samples, respectively. These data demonstrate relatively good discrimination between the control and SPC-fed rats in both regions. Corresponding loadings plots in Fig. S2(b,d) clearly reveal the highest loading peaks responsible for group separation in both the stem and cerebellum tissue samples, respectively. The *m/z* 184 (phosphatidylcholine headgroup [C_5_H_15_PO_4_N]^+^) is more closely associated with the SPC-fed group and *m/z* 369 (cholesterol [C_27_H_45_]^+^) is more associated with the control group, and these are among the highest loading peaks. This correlates well with what is observed for PCA analysis in whole tissue samples. Also for the negative data, different ROIs containing the stem and cerebellum part of the brain tissue were also treated separately. Similarly, as seen in representing score and loading plots shown in Fig. S3(a–d), the groups are separated in both the stem and cerebellar regions mainly by changes in as *m/z* 140 (phosphatidylethanolamine), *m/z* 180 (phosphinothricin), *m/z* 255 (palmitic acid [C_16_H_31_O_2_]^−^), *m/z* 281 (oleic acid [C_18_H_33_O_2_]^−^), and *m/z* 385 (cholesterol [C_27_H_45_O]^−^) and *m/z* 429 (Vitamin E [C_29_H_50_O_2_]^−^). The control group is associated with *m/z* 385 (cholesterol [C_27_H_45_O]^−^) and *m/z* 429 (Vitamin E [C_29_H_50_O_2_]^−^), whereas the SPC-fed group is associated with *m/z* 140 (phosphatidylethanolamine), *m/z* 180 (phosphinothricin), *m/z* 255 (palmitic acid [C_16_H_31_O_2_]^−^) and *m/z* 281 (oleic acid [C_18_H_33_O_2_]^−^) in both the stem and cerebellum regions of the brain. The results obtained here correspond to what is observed in whole tissue analysis. Therefore, we can conclude that from evaluation of the separate regions of the stem and cerebellum that different types of lipids do not change differently in different regions of interest, even though they change from the SPC diet overall.

### Statistical verification of the comparison between SPC-fed and control animals

**Figure 4:**
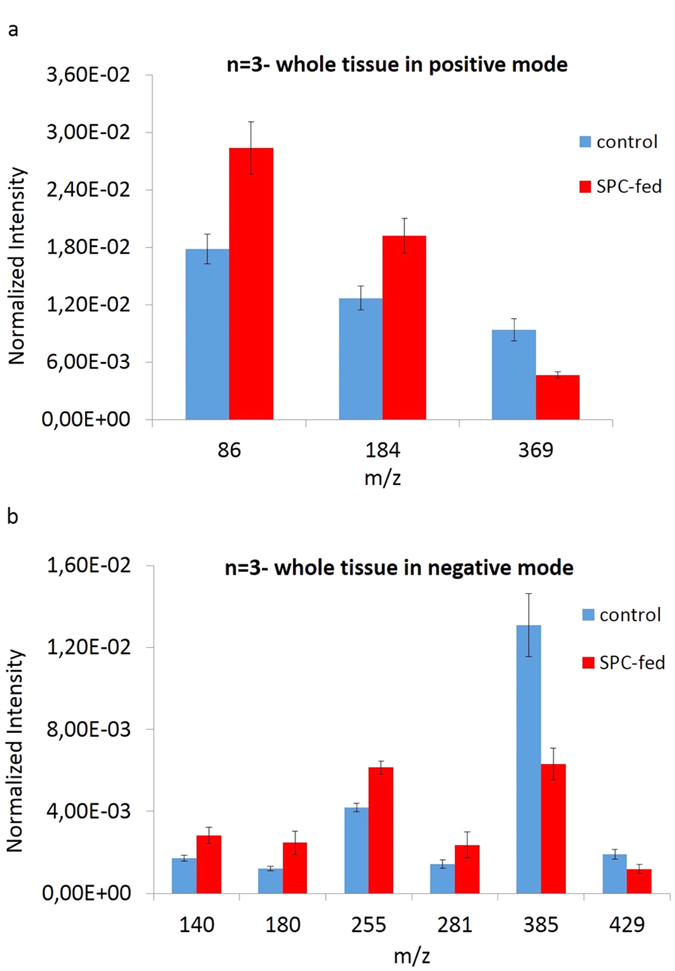
(**a**) Normalized intensity for positive species at *m/z* 86 (PC: C_5_H_12_N), *m/z* 184 (phosphatidylcholine head group) and *m/z* 369 (cholesterol) in control (blue) and SPC-fed (red) rat brains. (**b**) Normalized intensity of negative ions at *m/z* 140 (phosphatidylethanolamine)*, m/z* 180 (phosphinothricin), *m/z* 255 (palmitic acid), *m/z* 281 (oleic Acid), *m/z* 385 (cholesterol) and *m/z* 429 (Vitamin E) in control (blue) and SPC-fed (red) rat brains. In both charts, the bar heights show the average value and the error bars show 95% confidence level for each lipid peak in 3 animals containing 15 brain tissues for each control and SPC-fed sample.

To test the validity of the PCA models, the highest loading peaks causing separation between two groups of rats with different feeds were found from the loading plots illustrated in Figs(2b and 3b) for positive and negative ions, respectively, and were then analyzed semi-quantitatively with a t-test. The average values of these peaks are plotted, tested according to (*p* ≤ 0.05), and shown in Fig. 4(a) for positive and (b) for negative data.

Based on what was observed in Fig. 4(a), positive ions at *m/z* 86 (PC: C_5_H_12_N) and *m/z* 184 (phosphatidylcholine headgroup [C_5_H_15_PO_4_N]^+^) increase, whereas the ion *m/z* 369 (cholesterol [C_27_H_45_]^+^) decreases in the SPC-fed group. This matches well with the related loadings plot shown in Fig. 2(b) in which *m/z* 86 (PC: [C_5_H_12_N]^+^) and *m/z* 184 (phosphatidylcholine headgroup [C_5_H_15_PO_4_N]^+^) are associated with the SPC-fed group and *m/z* 369 (cholesterol [C_27_H_45_]^+^) is associated with the control group.

In the negative ion mode, Fig. 4(b) clearly indicates that *m/z* 140 (phosphatidylethanolamine), *m/z* 180 (phosphinothricin), *m/z* 255 (palmitic acid [C_16_H_31_O_2_]^−^), and *m/z* 281 (oleic acid [C_18_H_33_O_2_]^−^) ions are increased in the SPC-fed group, whereas *m/z* 385 (cholesterol [C_27_H_45_O]^−^) and *m/z* 429 (Vitamin E [C_29_H_50_O_2_]^−^) are reduced in the SPC-fed group. This is expected based on what is observed in the corresponding loadings plot illustrated in Fig. 3(b) in which the control group appears to be associated with *m/z* 385 (cholesterol [C_27_H_45_O]^−^), and *m/z* 429 (Vitamin E [C_29_H_50_O_2_]^−^), whereas the SPC-fed region is mostly associated with the fatty acids.

### Correlation of PCA data with TOF-SIMS images

In order to examine our findings in PCA analysis on spectra, some highest loading peaks indicated by PCA loadings plot in negative mode [Fig. 3(b)] such as *m/z* 180, 281, 385 and 429 were selected and imaged across the tissue sections in both control and SPC-fed groups. These secondary ion images were then normalized to the total ion counts and are shown in Fig. S4(a–h). The images show a slight increase in spatial signal intensity of ions such as *m/z* 180 and 281 in SPC-fed tissue whereas the signal intensity of the two ions of *m/z* 385 and 429 are decreased in SPC-fed tissue section which corresponds well with what is observed in related PCA loading plot [Fig. 3(b)] and the statistical data analysis of the spectra also [Fig. 4(b)]. But since PCA and statistical data analysis were performed on the 3 rats containing 15 sections in both control and SPC-fed groups, the changes are more obvious than in the images belonging to one tissue section per each group.

### Functional lipid composition in the brain is diet related

According to the results in this study the lipid species, cholesterol and Vitamin E, decrease whereas phosphatidylcholine (PC), phosphatidylethanolamine (PE), phosphinothricin, and fatty acids such as palmitic and oleic acid increase in the rat brain after feeding them SPC diet. These lipid species are all biologically important in cellular function.

After white adipose tissue, the brain is the organ most enriched with lipid compounds in the body^16^. Therefore, it is obvious that any systematic lipid alteration can heavily influence the brain structure and function. It is well established that many neurological diseases are associated with lipid perturbation in the brain^21^,^23^,^38^. The brain mainly consists of three major categories of lipids namely cholesterol, glycerophospholipids (phosphatidylcholine, phosphatidylethanolamine, and phosphatidylinositol) and sphingolipids (sphingomyelin, cerebrosides, sulfatides, and gangliosides)^15^. Notably, cholesterol and glycerophospholipids are the main lipid constituents in the lipid bilayer of cell membranes in the brain tissue such that 20% of the body’s total cholesterol exists in the brain and also 20–25% of the dry weight of the brain is comprised of glycerophospholipids^39^,^40^,^41^.

Surprisingly, cholesterol is one of the lipid species that we found to decrease in brain sections of SPC-fed rats. This lipid species is the most abundant lipid component in the white matter forming the insulating myelin sheath and plays a key role in more efficient transmission and rapid conduction of electrical impulses in the neuronal tissue^41^,^42^. Within the cell membrane, cholesterol increases membrane rigidity through the interaction with phospholipid fatty-acid chains and also in this structural role, cholesterol modulates membrane fluidity in different physiological temperatures^43^. Another function of cholesterol is the reduction of the plasma membrane permeability to neutral solutes, hydrogen ions and sodium ions^44^,^45^. Moreover, recent studies suggest a role for cholesterol in assisting with sphingolipids to form lipid raft regions which seem to be the residential locations of transmembrane proteins and ion channels in plasma membrane^46^. The obvious speculation is that decreasing cholesterol following SPC feeding, might facilitate cellular function making the cell membranes less rigid, more permeable, and changing lipid rafts. A lot more work would be needed to prove these concepts.

Our findings show both PC and PE lipid species are increased after using the SPC diet. Both PC and PE are from the main category of glycerophospholipids, which is one of the main constituents in the lipid bilayer of cell membranes, specifically in brain tissue. However, due to their geometries they have different localizations through the cell membrane, such that the lamellar (cylindrical) shaped PCs are distributed in the outer leaflet of cell membranes. In contrast, conical shaped lipid species like PE are mainly localized in the inner leaflet of the lipid bilayer. Consequently, the different geometries and localizations lead to different lipids serving different regulatory tasks in different cellular processes. For example during the exocytosis process, conical lipids such as PE mainly accumulate in high curvature regions within the cell membrane, whereas the lamellar shape PC lipid components are mainly rearranged in lower curvature spots of the lipid bilayer to assist and facilitate this cellular fusion^47^,^48^. Accordingly, distribution, structure and composition of lipids are of particular importance to maintain membrane integrity and to regulate substance flow across the ion channels within the plasma membrane^49^. Correspondingly, a supporting study showed that cell inflammation might be initiated by a reduction in membrane potential due to decrease in ratio of PC to PE in the cell membrane^18^. In addition, different cell types have distinctive and constant profiles of phospholipid species and any perturbation here has been found to lead the cell death^50^. Furthermore, PE the second most abundant phospholipid in the cell membrane, is the backbone in most living cell membranes, has been also recognized as the donor of the ethanolamine moiety to provide covalent attachment for signaling proteins in the plasma cell membrane^51^. As PC and PE are different in their lamellarity, it is a little difficult to speculate the function as they both increase after SPC feeding. It might be that this increase is localized to where the membranes are more flat (PC) versus highly curved as in a fusion pore (PE) and this might facilitate processes from exocytosis to anti inflammation.

Palmitic acid as well as oleic acid is increased in the SPC-fed group. These two lipid species are unsaturated fatty acids (FA), one of eight main groups of lipid components in living organisms^52^. Basically, phospholipids are the fundamental building blocks in the cell membrane and consist of two hydrophobic FA tails linked to a hydrophilic head phosphate group and FAs have been well found to modify membrane structure and function. In this regard, the effect of oleic acid on permeability of blood-brain barriers has been showed previously^53^. In addition, FAs are more easily reduced yielding more energy than carbohydrates and proteins, in general these lipid components are well known as main reservoirs of energy for biological systems^54^.The increase in these fatty acids following SPC feeding might act merely to increase the source of energy in the brain, but might have structural effects as well. This leaves open some serious study for the future and might include omega 3 vs. 6 fatty acids and their effect on cell and brain function.

Our findings demonstrate that changes occur in biologically relevant lipid components in the cell membrane following the SPC cereal feeding. This observed structural membrane alternation might be involved in the working mechanism of a SPC-induced AF activation in neuronal tissue and might be good evidence for recent studies localizing this endogenous protein followed by SPC diet in the central nervous system^35^,^36^.

The data obtained and presented in this work indicate that changes of certain lipids occur in rat brain tissue following consumption of SPC cereals. PCA score plots show a well-defined separation in the lipid distribution between the control and the SPC-fed groups based on ToF-SIMS data. In the positive ion mode, based on the related loading plot, the two groups are overall well separated from each other owing to changes in phosphatidylcholine and cholesterol. The loading plots reveal that the groups observed in the negative ion mode are separated mainly due to changes in the cholesterol, Vitamin E and c18:1, c16:0 fatty acid distribution as well as short chain monocarboxylic fatty acids. The data have also been analyzed semi-quantitatively with the Welch’s t-test to validate the findings from the multivariate analysis. Overall, the SPC-fed group is associated with higher levels of fatty acids and phosphatidylcholine and lower levels of cholesterol and Vitamin E than the control group. The results are consistent regardless of which histological region, brain stem or cerebellum, is analyzed. Hence, this study shows that ToF-SIMS can serve as an analytical tool to track food induced changes in lipid content in brain tissue. We also demonstrated the involvement of several important cell membrane lipid species in response to SPC intake. This indicates that AF might form peptide-cell interactions with the cell membrane through the peptide exposure of active sites, thus supporting recent studies^2^,^5^,^55^ on the ability of this feed to induce the endogenous production of AF in the body. The results might have significance to understanding the basic function of lipids in brain and cell function as well.

## Methods

### Animals and diets

The experimental design was approved by the Regional Animal Experiments Ethical Committee in Gothenburg, Sweden. All experiments were performed according to guidelines for animal experiments (EC Directive 86/609/EEC). Male Sprague-Dawley rats displayed a body weight of 180 ± 20 g, and the rats were allowed a week for general adaption in their cages before change of feed started. All rats had free access to water and pelleted food during the whole experimental period.

In order to stimulate the endogenous formation of AF, a diet of 10% specially processed cereals was mixed to standard rat feed (Lab For Rat 36; Lantmännen). The mixed feed was pelleted and processed in the same way as the R36 feed. The quality of both batches of experimental and control were coded and then checked based on Lantmännen standard procedures. The composition of the feed was taken from the work of Johansson *et al*.^56^ The animals in both the control and experimental groups had free access to water and food during the four weeks long experimental period.

### Tissue preparation

After the four weeks long diet period, the rats were brought to deep anesthesia by Isoflurane inhalation (4% induction, 1.5–2% maintenance in air) followed by heart puncture with a needle (OD 1.2 mm, length 60 mm) and bleeding the rat of some 10–12 ml blood. The thorax was then opened and the heart was removed. Subsequently, the skull was opened and then the brain hemispheres, the small brain, and the brain stem were carefully dissected out and immediately frozen in liquid N2 for the following processing of sections for imaging ToF-SIMS. All of the cryosections prepared from various parts of the brain were 10 μm thick and mounted onto ITO glass slides. The slides were then placed in closed containers and stored at −80 °C. Prior to TOF-SIMS analysis, they were warmed to room temperature and then dehydrated in a desiccator under vacuum for 30 min.

### ToF-SIMS Analysis

The ToF-SIMS experiments were carried out with a ToF.SIMS 5 instrument (ION-ToF GmbH, Münster, Germany) equipped with a liquid metal primary ion source. ToF-SIMS spectra of positive and negative ions were recorded using Bi_3_^+^ primary ions at 25 keV energy in high current bunched mode (mass resolution m/∆m = 5000)^57^ with a pulsed primary ion current of 0.25 pA as well as electron flooding for charge neutralization. The maximum ion dose density was maintained around 1.5 × 10^11^ ions cm^−2^ during all experiments, below the static limit (1 × 10^13^ ions cm^−2^), to minimize surface damage. The images were recorded in an area of approx. 11 × 11 mm covering the whole tissue sections with lateral resolution of 5 μm. In total, 3 different animals from each group with 5 technical repeats were analyzed.

All ToF-SIMS data analyses were performed with the Surface Lab software (version 6.3 ION-ToF, GmbH, Münster, Germany). The spectra were internally calibrated to signals of [C]^+^, [CH]^+^, [CH_2_]^+^, [CH_3_]^+^, [C_5_H_15_PNO_4_]^+^ and [C_27_H_45_]^+^ that are commonly used for calibration in positive and [C]^−^, [CH]^−^, [C_2_]^−^, [C_3_]^−^, [C_16_H_31_O_2_]^−^, and [C_18_H_33_O_2_]^−^ in negative ion mode. Specific regions of interest (ROI) containing the whole tissue, the stem, and the cerebellum regions of the brain samples were separately created. The search peaks function in Surface Lab software was employed on spectra obtained from the individual samples to select peaks according to the following search parameters: counts >100, S/N > 3, width 0.8 Da. The most intense and most significant peaks were collected, creating two mass interval lists (one for each ion polarity) with m/z values to be included in the multivariate analysis. Data including a set of 453 (negative ions) and 696 (positive ions) obtained from the brain samples were normalized to the total ion counts of the respective spectra and then subjected to PCA data analysis using SIMCA (version 13.0, Umetrics, Umea, Sweden). Here the spectra were pretreated by Mean-centering and Pareto scaling. In order to remove redundant and not chemically specific peaks such as hydrocarbon clusters, low-mass peaks (below *m/z* = 100 for negative and *m/z* = 60 for positive ions) were eliminated from the PCA. Subsequently, the scores and loading plots extracting information as to which ions (*m/z* values) are responsible for the majority of the variation between the two groups, control and SPC-fed rats, were generated from the PCA model. Ultimately, the lipid species that significantly changed between two groups of control and SPC-fed were found by t-test analysis and then identified according to previous reported literature^58^,^59^.

## Additional Information

**How to cite this article**: Pour, M. D. *et al*. Food-induced changes of lipids in rat neuronal tissue visualized by ToF-SIMS imaging. *Sci. Rep.* **6**, 32797; doi: 10.1038/srep32797 (2016).

## Supplementary Material

Supplementary Information
