# Supplementary material for: Food-induced changes of lipids in rat neuronal tissue visualized by ToF-SIMS imaging
Source: Sci Rep. 2016 Sep 6;6:32797. doi: 10.1038/srep32797 (PMC5011716; doi:10.1038/srep32797)
Supplement: Supplementary Information [file srep32797-s1.doc]

**Food-induced changes of lipids in rat neuronal tissue visualized by**

**ToF-SIMS imaging**

| Masoumeh Dowlatshahi Pour^1,2^; Eva Jennische^3^; Stefan Lange^3^;  Andrew Ewing^1,2,4^, Per Malmberg^1,2^. |
| --- |
| ^1^Department of Chemical and Biological Engineering, Chalmers University of Technology, Gothenburg, Sweden  ^2^National center for imaging mass spectrometry, Gothenburg, Sweden  ^3^Institute of Biomedicine, Gothenburg, Sweden;  ^4^ Department of Chemistry and Molecular Biology, University of Gothenburg, Gothenburg, Sweden  **In this Supporting Information, we present optical microscopy images of freeze-dried rat brain sections for both groups of control and SPC-fed, as well as ToF-SIMS images showing the spatial signal intensity distribution from negative ions of *m/z* 140 and 255 and total ion in both groups of control and SPC-fed, PCA scores and loadings bi plots of positive and negative modes of ToF-SIMS in both stem and cerebellum tissue and ToF-SIMS ion images normalized to total ion counts from highest loading peaks in negative mode; the ions of m/z 180, 281, 385 and 429 in both groups of control and SPC-fed.** |


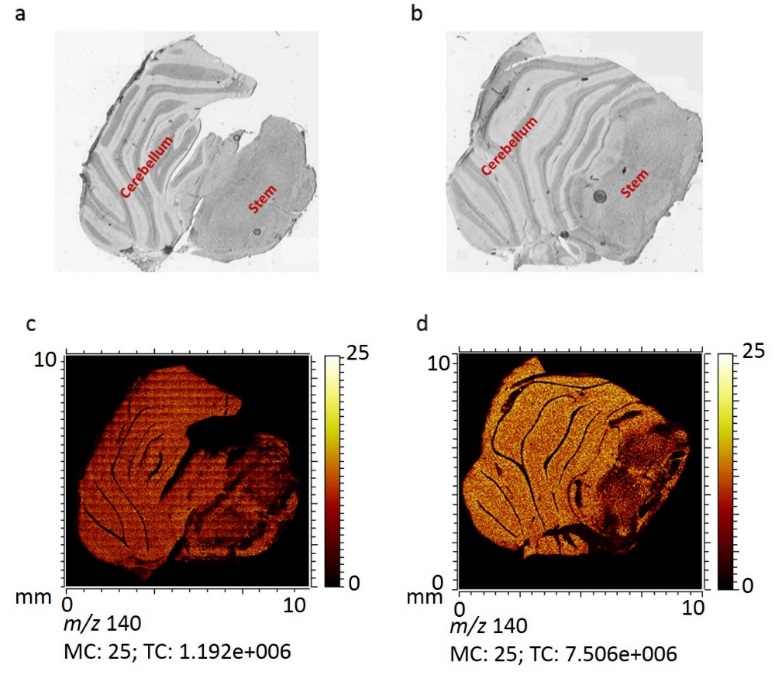

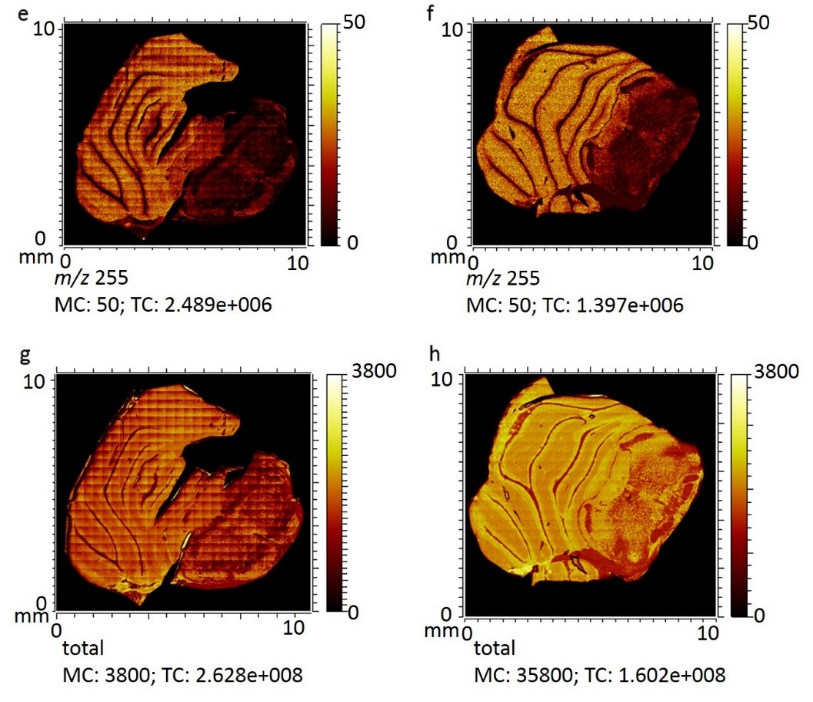


Fig.S1. Optical microscopy images of freeze-dried rat brain sections brain section containing cerebellum and stem are shown in (a) control and (b) the SPC-fed group. ToF-SIMS images showing the spatial signal intensity distribution from negative ions are shown for *m/z* 140 (Phosphatidylethanolamine) (c) control and (d) the SPC-fed group, for *m/z* 255 (Palmitic acid) in (e) control and (f) the SPC-fed group, and for total ions in (g) control and (h) the SPC-fed group across an analysis area of 11 × 11 mm covering the complete tissue.


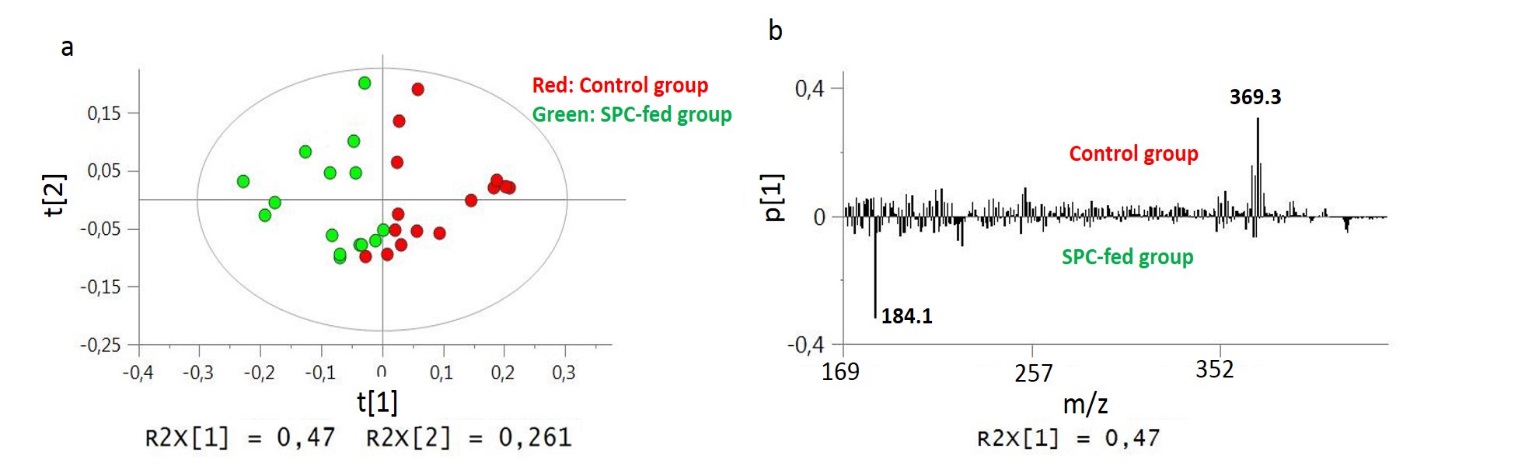


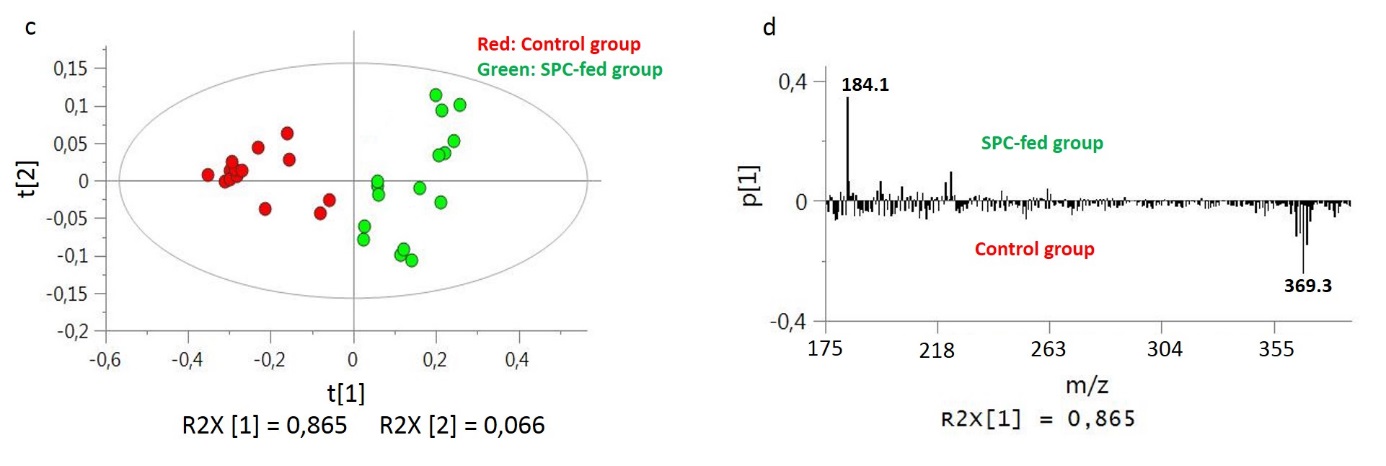


Fig.S2. Multivariate analysis of positive mode data of ToF-SIMS experiment from 3 rats containing 15 brain tissues for each SPC-fed and control ones (a) Scores plot of the first principle component (t[1]) vs. the second principle component (t[2]) of spectra related to stem tissue. (b) Corresponding loading plot of the first principle component (p[1]) showing the most responsible m/z peaks for the separation between groups related to stem tissue. (c) Scores plot of the first principle component (t[1]) vs. the second principle component (t[2]) of spectra related to cerebellum tissue. (d) Corresponding loading plot of the first principle component (p[1]) indicating the m/z peaks with strong impact on the separation between groups related to cerebellum tissue.


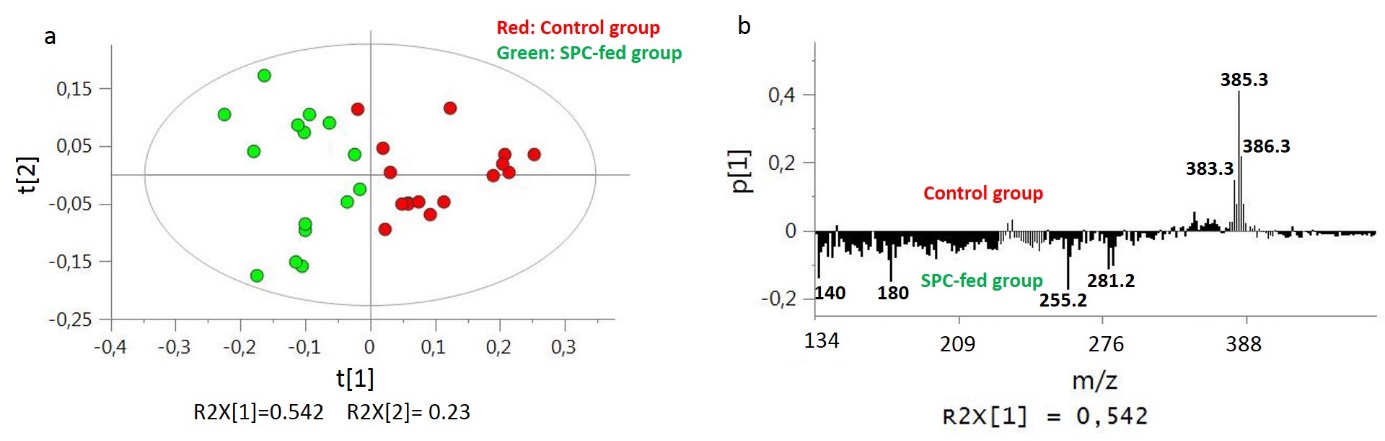


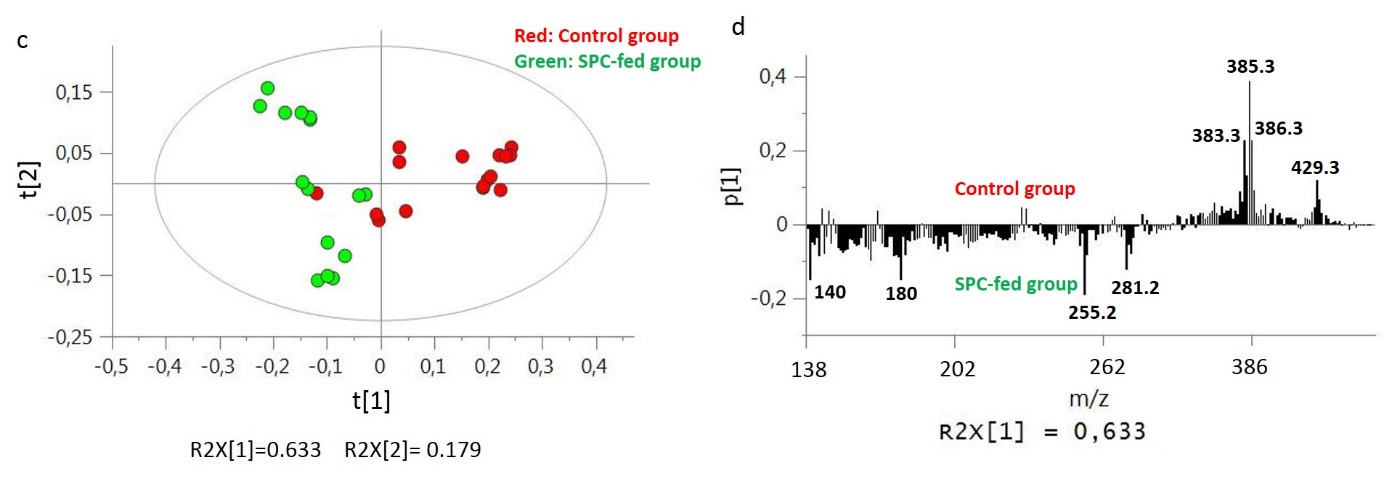
 Fig.S3. Multivariate analysis of negative mode data of ToF-SIMS experiment from 3 rats containing 15 brain tissues for each SPC-fed and control ones (a) Scores plot of the first principle component (t[1]) vs. the second principle component (t[2]) of spectra related to stem tissue. (b) Corresponding loading plot of the first principle component (p[1]) showing the most responsible m/z peaks for the separation between groups related to stem tissue. (c) Scores plot of the first principle component (t[1]) vs. the second principle component (t[2]) of spectra related to cerebellum tissue. (d) Corresponding loading plot of the first principle component (p[1]) indicating the m/z peaks with strong impact on the separation between groups related to cerebellum tissue.


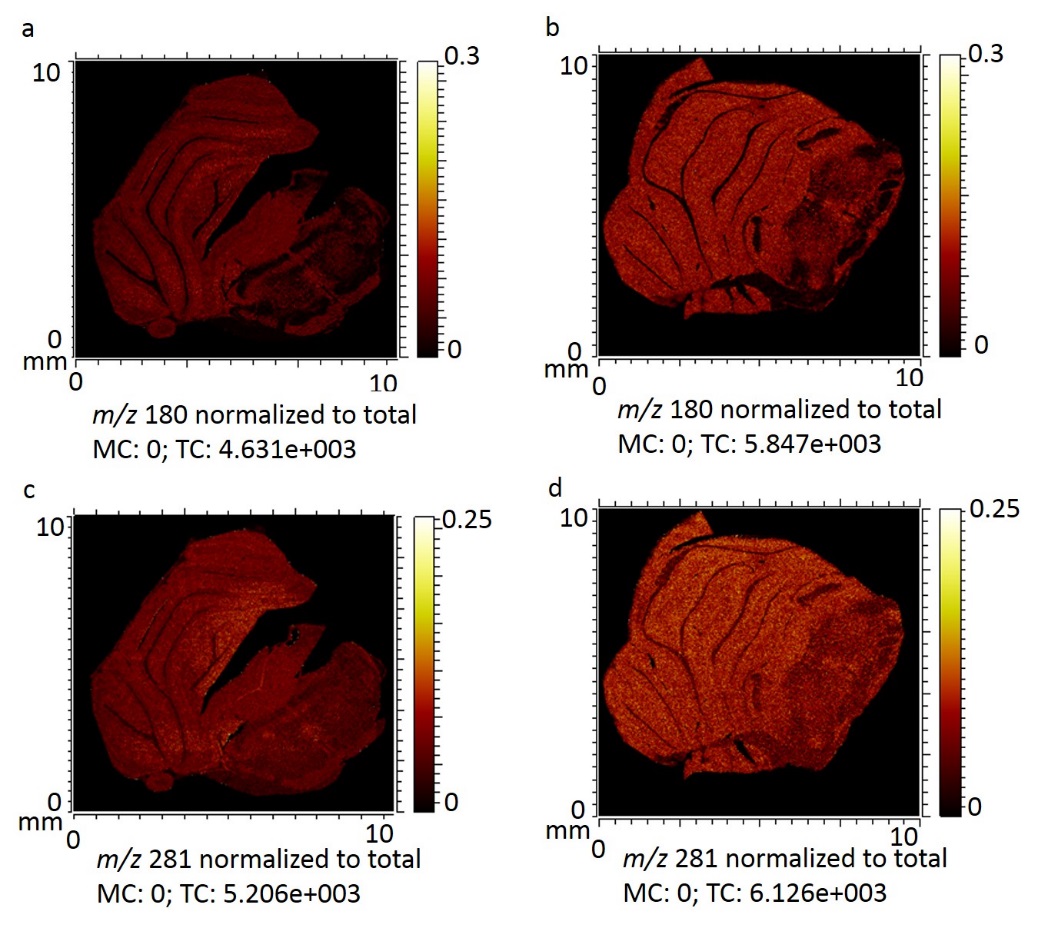

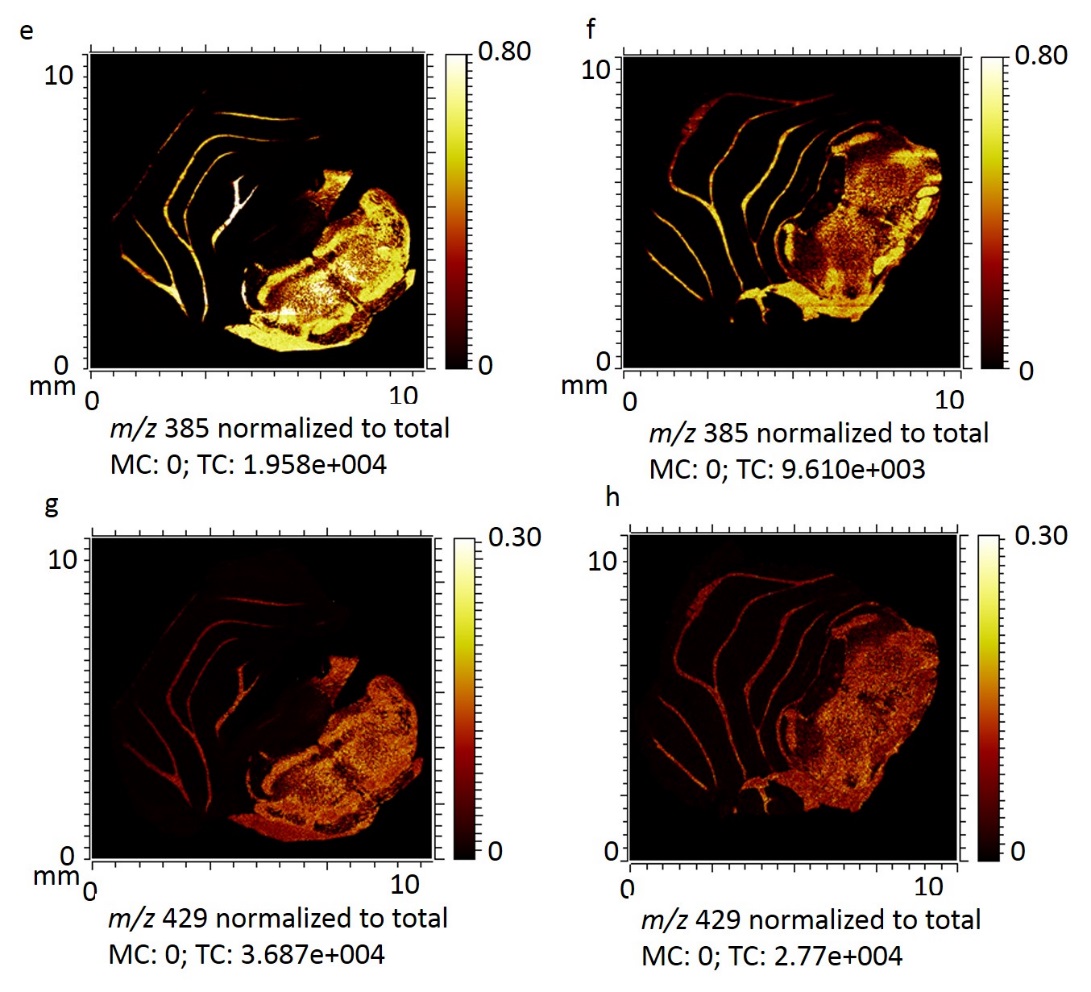


Fig.S4. ToF-SIMS images normalized to total ion counts from negative ions of highest loading peaks such as *m/z* 180 (phosphinothricin) in (a) control and (b) the SPC-fed group, *m/z* 281 (oleic acid) in (c) control and (d) the SPC-fed group, *m/z* 385 (cholesterol) in (e) control and (f) the SPC-fed group, and *m/z* 429 (Vitamin E) in (g) control and (h) the SPC-fed group across an analysis area of 11 × 11 mm covering the complete tissue.
